# Characterization of spontaneous spheroids from oral mucosa-derived cells and their direct comparison with spheroids from skin-derived cells

**DOI:** 10.1186/s13287-019-1283-0

**Published:** 2019-06-24

**Authors:** Ni Li, Xianqi Li, Kai Chen, Hongwei Dong, Hideaki Kagami

**Affiliations:** 1 Department of Hard Tissue Research, Graduate School of Oral Medicine, Matsumoto Dental University, Shiojiri, Japan; 2 Institute for Oral Science, School of Dentistry, Matsumoto Dental University, Shiojiri, Japan; 3 Department of Oral and Maxillofacial Surgery, School of Dentistry, Matsumoto Dental University, Shiojiri, Japan; 4 Department of General Medicine, IMSUT Hospital, The Institute of Medical Science, The University of Tokyo, Tokyo, Japan

**Keywords:** Oral mucosa, Skin, Somatic stem cells, Spontaneous spheroid, Pluripotent stem cells, Cell differentiation

## Abstract

**Background:**

Our group has developed a novel method for spontaneous spheroid formation using a specific low-adherence culture plate with around 90° water contact angle. In this study, this method was applied for oral mucosa-derived cells. First, the feasibility of spontaneous spheroid formation was tested. Next, the characteristics of spontaneous spheroids from oral mucosa- and skin-derived cells were compared with special focus on the stemness and neuronal differentiation capability.

**Methods:**

Oral mucosal cells were obtained from the palate and buccal mucosa of C57BL/6J mice. Similarly, skin cells were obtained from the back of the same mouse strain. Passage 2–3 cells were inoculated into the specific low-adherence culture plates to form spontaneous spheroids. The effect of basic fibroblast growth factor (bFGF), epidermal growth factor (EGF), and B27 supplement on spheroid formation and maintenance was assessed. Immunofluorescence and quantitative reverse transcription polymerase chain reaction (qRT-PCR) were performed to investigate the expression of pluripotency markers, cell proliferation and apoptosis markers, and neurogenic differentiation markers.

**Results:**

Using this culture plate, spontaneous spheroid formation was feasible. This process depended on the presence of serum but was independent of the additives such as bFGF, EGF, and B27 supplement, although they improved the efficiency and were essential for spheroid maintenance. This result was confirmed by the higher expression of Caspase7 in the spheroids cultured without the additives than that with the additives. The spheroids from oral mucosa-derived cells expressed stem cell markers, such as Sox2, SSEA1, Oct4, Nanog, and Nestin. The expression of Sox2 in spheroids from oral mucosal cells was higher than that in spheroids from skin-derived cells. Both spheroid-forming cell types had the ability to differentiate into neural and Schwann cells after neurogenic induction, although significantly higher MAP 2, MBP, Nestin, and Nurr1 gene expression was noted in the cells from oral mucosa-derived spheroids.

**Conclusions:**

The results showed that spontaneous spheroids from oral mucosa-derived cells contain highly potent stem cells, which were as good as skin-derived stem cells. The high expression of certain neuronal marker genes suggests an advantage of these cells for regeneration therapy for neuronal disorders.

**Electronic supplementary material:**

The online version of this article (10.1186/s13287-019-1283-0) contains supplementary material, which is available to authorized users.

## Background

Spheroid formation has been used for selective culture of stem cells from various tissues, including neuron, skin, salivary gland, bone marrow stroma, periodontal ligament, and dental pulp tissues [1–5]. Various methods are used to form spheroids. The hanging drop method utilizes a drop of cell suspension, and the cells start to aggregate at the bottom of the droplet via gravity, eventually forming a spheroid [6]. Rotation of culture medium has also been used [7, 8]. Because these methods depend on physical force and the cell-to-cell attachment is achieved by forced contact of the cells, we designated them as “mechanical spheroid formation” methods, and the process of spheroid formation is not selective for stem cells [9].

Other methods, including ours, do not use physical force, and spheroid formation occurs under static conditions. Accordingly, we designated these approaches as “spontaneous spheroid formation” methods [9]. Because spontaneous spheroid formation starts only from possible stem cells, it theoretically enables a selective culture of stem cells. Thus far, most reported spontaneous spheroid formation is limited to neural stem cells and skin-derived stem cells [10, 11], and the detailed characteristics of spontaneous spheroids from oral mucosa-derived cells have not been reported.

Among the cell sources for somatic stem cells, oral mucosa is considered very unique. Cell lineage studies have revealed that even adult tissues contain neural crest cell-like stem cells [12, 13]. Although oral mucosa and skin fibroblasts have a similar morphology and function, some essential differences exist. For example, wound healing in the oral mucosa is faster, with less scar formation compared with that in the skin [14, 15]. These characteristic features of oral mucosa might be due to the presence of highly potent neural crest-derived cells [16]. Accordingly, some researchers believe that oral mucosa would be a favorable source for somatic stem cells [17]. To support this idea, several recent studies have reported that the stem cell populations in the lamina propria of the adult lip and oral mucosa demonstrate neural crest-like characteristics and have exceptionally broad differentiation capacity, including differentiation into osteoblasts, chondroblasts, adipocytes, and neural lineages [18–21]. However, to the best of our knowledge, no studies have directly compared stem cells from the skin and oral mucosa.

In the present study, the feasibility of spontaneous spheroid formation from oral mucosa-derived cells was tested. Next, the roles of basic fibroblast growth factor (bFGF), epidermal growth factor (EGF), and B27 supplement, which are reported as essential factors for neurosphere formation, on spontaneous spheroid formation and maintenance were investigated. Finally, the characteristics of spontaneous spheroids from oral mucosa- and skin-derived cells were compared with special focus on their stemness and neural differentiation capabilities.

## Materials and methods

### Cell culture

The animal experiments were approved by the Matsumoto Dental University Committee on Intramural Animal Use (no. 289). Primary culture cells were obtained using a conventional explant culture technique. Mice (3–4 weeks old) were put to death with an anesthesia overdose, and then, the buccal and palatal mucosa and back skin were removed and cultured. The basic culture medium was αMEM (Wako Pure Chemical Industries, Ltd., Osaka, Japan) supplemented with 10% fetal bovine serum (FBS; Biowest, Nuaillé, French), 100 U/ml penicillin, 100 μg/ml streptomycin, and 0.25 μg/ml amphotericin B (Biological Industries USA, Inc., Cromwell, CT, USA). The cells were passaged at 80–90% confluence. The medium was changed every 3 to 4 days. Cells at passages 2–3 were used for the experiments.

The cultivation of compact bone-derived cells (CBDCs) was based on the protocol as reported in our previous study with modifications [9]. Briefly, the femurs and tibiae were dissected, the epiphyses were cut, and the bone marrow was flushed out. Then, the cortical bones were chopped into fine pieces and incubated in collagenase solution for 45 min at 37 °C in a bio-shaker. The cells were collected and cultured in the basic culture medium supplemented with 10 ng/ml basic fibroblast growth factor (bFGF) (PeproTech, Rocky Hill, USA). The bone chips were seeded with the same culture medium into the other well of the same dish to collect additional cells.

### Spheroid formation

The method used for spheroid formation was described in our previous publication [9]. Briefly, monolayer culture cells were trypsinized, and the cells were seeded in a 55-mm dish (1.5 × 10^4^ cells/cm^2^) (Azunol®, #1-8549-02, AS ONE, Osaka, Japan) for spheroid formation with basal culture medium. The medium was changed every 3 days. To evaluate the spheroid-forming efficiency of oral mucosa- and skin-derived cells, the number and size of the spheroids were determined from 1 to 5 days in four parallel phase-contrast microscope fields per dish using a × 4 objective lens (Olympus IX70 inverted microscope, Olympus Optical CO, Ltd., Tokyo, Japan). To evaluate the maintenance of spheroids, basic fibroblast growth factor (bFGF; Gibco, Carlsbad, CA, USA), epidermal growth factor (EGF; PeproTech, Rocky Hill, NJ, USA), and B27 supplement (Gibco, Carlsbad, CA, USA) were added to the basal medium. To investigate the necessity of each factor, the culture was performed with one or some of these additives. The diameter and number of spheroids were determined for 5 days.

### Neurogenic differentiation

Spheroids from oral mucosa-, skin-, and CBDC-derived cells were transferred into a new regular culture dish. After these cultures reached 50–60% confluence, the medium was changed to neurogenic induction medium. The medium used for neural cell differentiation was αMEM supplemented with l-glutamine, phenol red (Wako), 50 ng/ml nerve growth factor, 50 ng/ml brain-derived neurotrophic factor, 10 ng/ml NT-3 (all from Peprotech), 10% FBS, 100 U/ml penicillin, 100 μg/ml streptomycin, and 0.25 μg/ml amphotericin B (Biological Industries). For Schwann cell differentiation, the medium used was αMEM supplemented with l-glutamine, phenol red (Wako), 5 μM forskolin (Sigma), 50 ng/ml heregulin-1β (Peprotech), 2% *v*/*v* N2 supplement (Invitrogen), 10% FBS, 100 U/ml penicillin, 100 μg/ml streptomycin, and 0.25 μg/ml amphotericin B (Biological Industries). The cells were differentiated for 1 or 2 weeks, and 50% of the medium was changed every 2 days.

### Immunofluorescence staining

Immunofluorescence staining was performed as previously reported [9]. Primary antibodies targeting the following proteins were used: SSEA1 (1:40, ab16285, Abcam), Sox2 (1:250, 97959, Abcam), Oct4 (1:250, ab19857, Abcam), Nanog (1:100, ab80892, Abcam), βIII-tubulin (1:250, ab87087, Abcam), Nestin (1:200, ab6142, Abcam), NEUN (1:100, ab177487, Abcam), MAP 2 (1:50, ab32454, Abcam), and S100β (1:100, ab52642, Abcam). The secondary antibodies used were as follows: goat anti-mouse IgM Alex Fluor 488 (1:200, ab150121, Abcam), goat anti-mouse IgG Alex Fluor 488 (1:500, ab150113, Abcam), and goat anti-rabbit IgG Alex Fluor 647 (1:200–1:500, ab150079, Abcam), and the nuclei were counterstained with 4′,6-diamidino-2-phenylindole (DAPI, ab104139, Abcam) for 30 min at room temperature.

To investigate the cell proliferation and apoptosis inside the spheroids, immunofluorescence staining for Ki67 and Caspase7 was performed. Spheroids were collected 3 days after seeding to the low adherent plate and solidified in iPGell (Genostaff, Tokyo, Japan) according to the manufacturer’s instructions, fixed with 4% paraformaldehyde in phosphate buffer, embedded in paraffin, and sectioned at a thickness of 8 μm. The sections were permeabilized and blocked with 5% BSA, 5% donkey serum, and 0.5% Triton X-100 in PBS for 1 h. Primary antibodies targeting the following proteins were used: Ki67 (1:100, ab15580, Abcam) and Caspase7 (1:100, ab69540, Abcam). After incubation with primary antibodies overnight at 4 °C, the sections were washed with PBS for 3 times and incubated with the respective secondary antibodies. The secondary antibodies used were as follows: donkey anti-mouse IgG Alex Fluor 488 (1:250, ab96875, Abcam) and donkey anti-rabbit IgG Alex Fluor 647 (1:250, ab150075, Abcam), and the nuclei were counterstained with DAPI solution.

The fluorescence was observed with a phase-contrast microscope (KEYENCE BZ-X710, Keyence, Osaka, Japan) and analyzed by BZ-X Analyzer software.

### Quantitative reverse transcription polymerase chain reaction

Total RNA was extracted from cells using TRIzol reagent (Invitrogen Corporation, Carlsbad, CA, USA), and cDNA was synthesized with PrimeScriptTM RT Master Mix (Perfect Real Time) (TaKaRa, cat# RR036A, Kusatsu, Japan) according to the manufacturer’s protocol. qRT-PCR was performed at least in triplicate according to the manufacturer’s protocol. The data were quantified using the ∆∆Cycle threshold method and were normalized against the levels of β-actin. The primer sequences used for PCR are provided in Table 1.

**Table 1:**
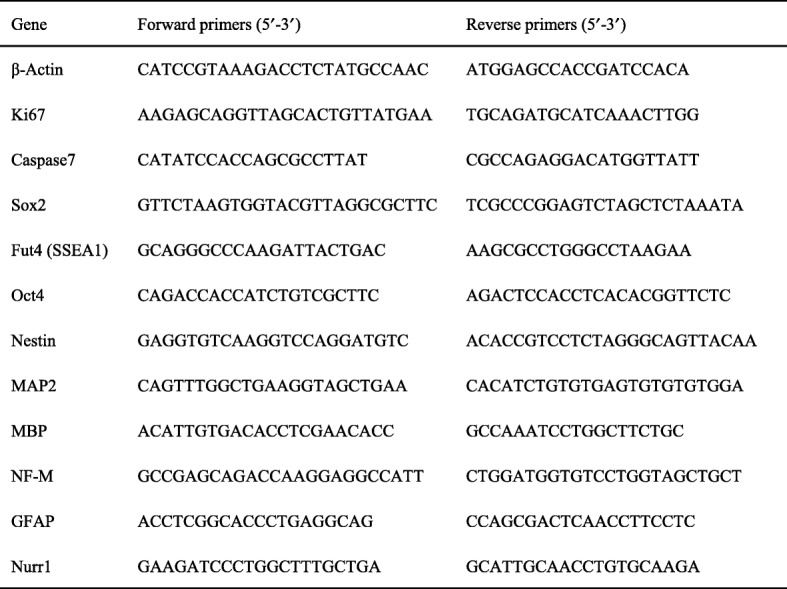
Primer sequences for PCR

### Statistical analysis

Statistical analysis was performed using Student’s *t* test. The results are presented as the mean ± standard error of the mean (SEM) of a minimum of three experiments.

## Results

### Spontaneous spheroids from oral mucosa-derived cells

**Fig. 1:**
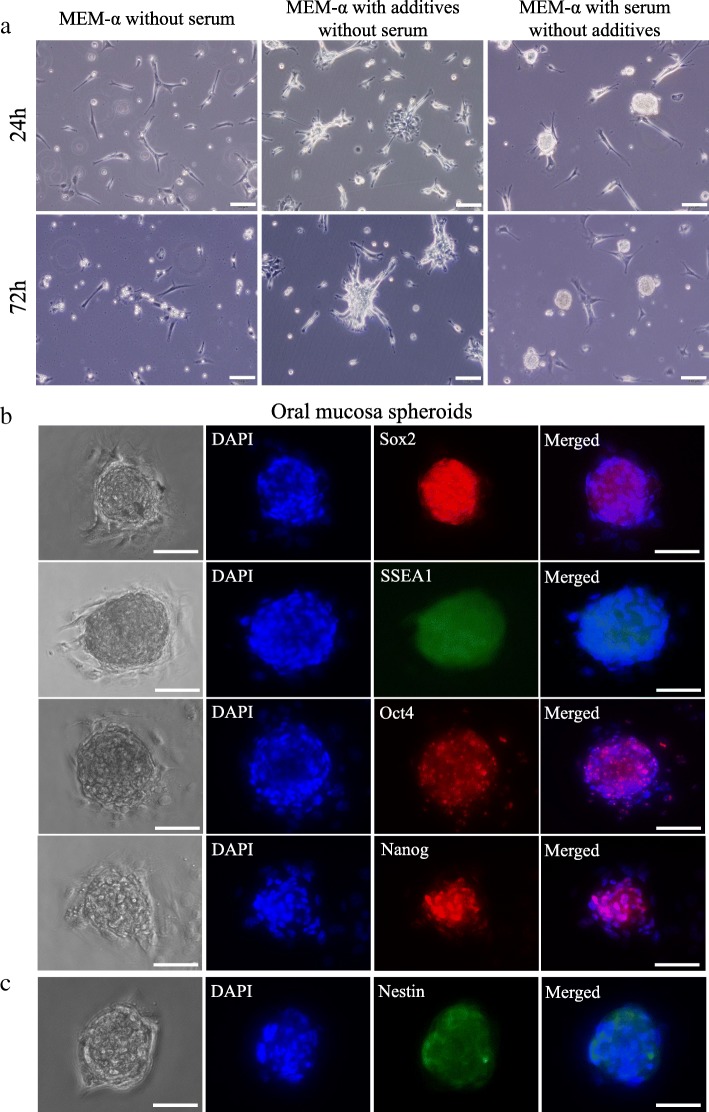
Characterization of spontaneous spheroids from oral mucosa-derived cells. **a** Morphological changes of oral mucosa-derived cells on the specialized culture dish. In serum-free culture medium and serum-free culture medium with bFGF, EGF, and B27 supplement, no spheroid formation was observed. Only cells in medium containing serum formed spheroids spontaneously, which were then maintained. Scale bars = 100 μm. **b** Immunofluorescence analysis of Sox2, SSEA1, Oct4, and Nanog expression in spheroids from oral mucosa-derived cells. A positive reaction was observed in almost all spheroid-forming cells. **c** The expression of Nestin in spheroids from oral mucosa-derived cells. Scale bars = 50 μm. DAPI, 4′,6-diamidino-2-phenylindole

The spheroid-forming ability of the oral mucosa-derived cells on the specific culture dish was examined. In serum-free culture medium, some of the oral mucosa-derived cells attached to the culture dish and showed fibroblastic morphology at 24 h, and then, the cells became apoptotic after 72 h (Fig. 1a). In serum-free culture medium containing bFGF, EGF, and B27 supplement, oral mucosa-derived cells attached to the dish and showed some cell aggregates, but spheroid formation was not observed (Fig. 1a). In medium containing serum, oral mucosa-derived cells formed spheroids spontaneously within 24 h, and the spheroids were maintained during the observation period (72 h) (Fig. 1a).

To examine the expression of stem cell marker genes, spheroid-forming cells were analyzed via immunofluorescence. The spontaneous spheroids formed from oral mucosa-derived cells expressed Sox2, SSEA1, Oct4, Nanog (Fig. 1b), and Nestin (Fig. 1c).

### Effect of additives on spheroid formation and maintenance of oral mucosa- and skin-derived cells

**Fig. 2:**
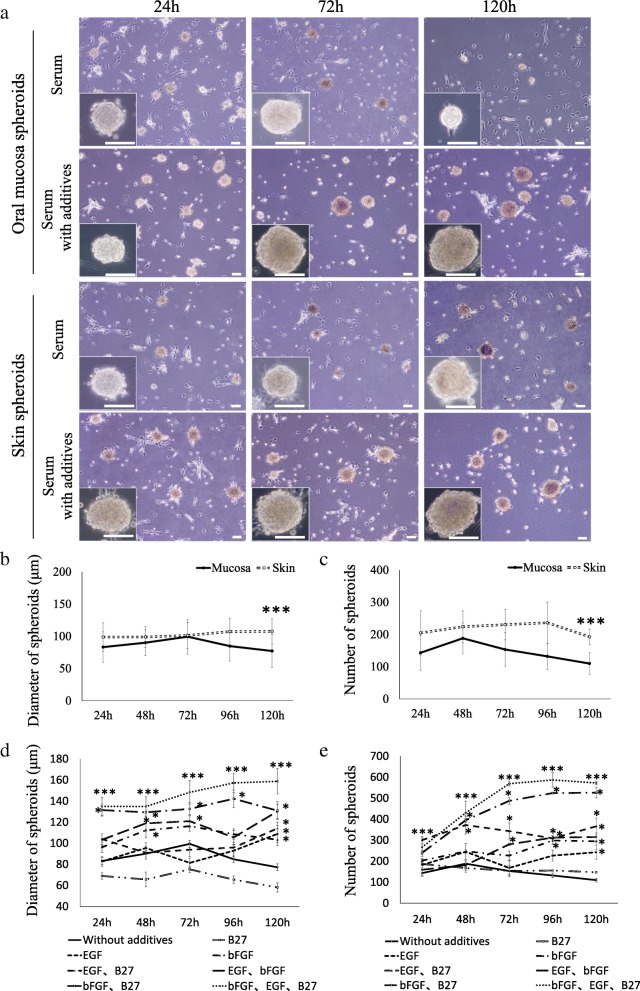
Spontaneous spheroid formation from oral mucosa- and skin-derived cells. **a** Phase-contrast images of spheroids. In medium containing serum, oral mucosa-derived cells and skin-derived cells spontaneously aggregate and form compact multicellular spheroids within 24 h after cell seeding. **b** The size of spheroids from oral mucosa- and skin-derived cells. The difference was significant at 120 h; *n* = 10. **c** The number of spheroids from oral mucosa- and skin-derived cells. A significant difference was observed only at 120 h; *n* = 10. **d** The effect of additives on the size of spheroids. Spheroid size was significantly larger in medium containing bFGF, EGF, and B27 supplement than in medium without these additives; *n* = 20. **e** The effect of additives on the number of spheroids. A larger number of spheroids were observed in medium containing bFGF, EGF, and B27 supplement than in medium without these additives; *n* = 6. Scale bars = 100 μm. The data are presented as the mean ± SEM; *p < 0.05; ***p < 0.001

In medium containing serum, the oral mucosa-derived cells and skin-derived cells spontaneously aggregated to form compact multicellular spheroids within 24 h without additives and were maintained for 72 h (Fig. 2a). Spheroid morphology was not affected by the presence of additives or the origin of the cells, and the difference between groups was not remarkable.

To further investigate the changes in spheroids during the time course, the diameter and number of spheroids were assessed for up to 120 h. The size of spheroids from oral mucosa-derived cells tended to increase for up to 72 h after cell seeding and then decreased gradually over time (Fig. 2b). On the other hand, the size of the spheroids from skin-derived cells did not change for up to 120 h, and the difference between the spheroids was significant at 120 h (*p* < 0. 001). The number of spheroids from oral mucosa-derived cells increased and peaked at 48 h and then reduced gradually (Fig. 2c). The number of spheroids from skin-derived cells gradually increased for up to 96 h and then decreased. The difference in number was significant only at 120 h (*p* < 0. 001).

Next, the diameter and number of spheroids were determined in the presence of one or some of the additives. Compared with the spheroids without additives, the spheroid size was significantly larger in medium containing bFGF, EGF, and B27 supplement (*p* < 0.001) (Fig. 2d). The closest results were obtained when the spheroids were cultured with bFGF and B27 supplement. The number of spheroids was also higher in the group cultured with all three additives from 24 to 120 h than in the groups cultured without additives (*p* < 0.001) (Fig. 2e). Similar results were obtained in the group treated with bFGF and B27 supplement, and the number was larger than that in the groups without additives at all time points.

### Analyses of cell proliferation and apoptosis inside the spheroids

**Fig. 3:**
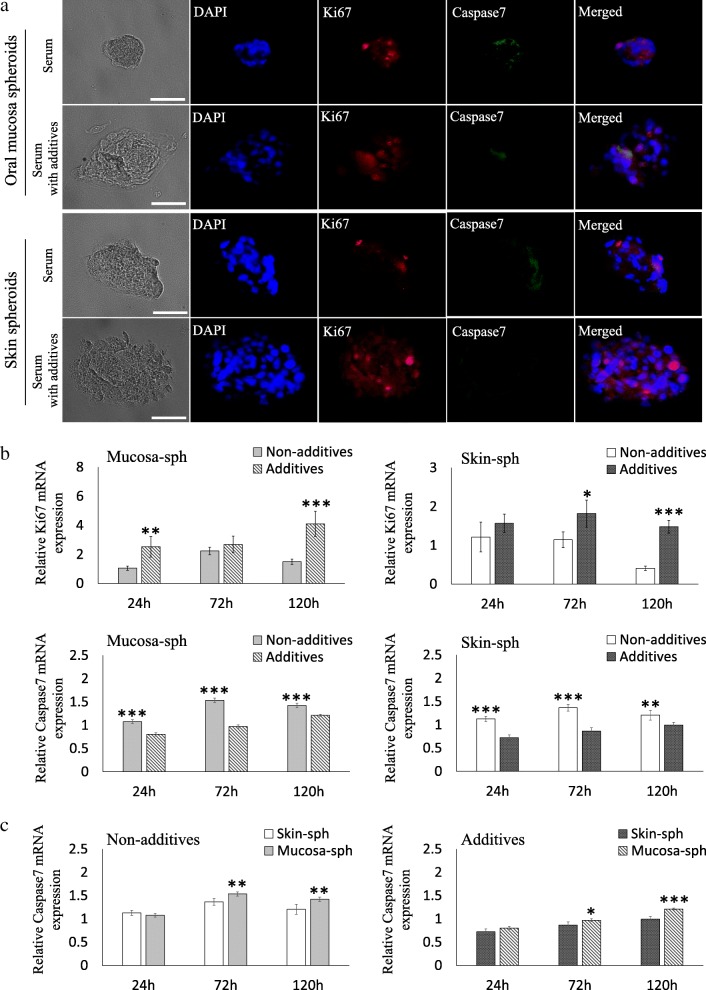
Cell proliferation and apoptosis in spheroids from oral mucosa- and skin-derived cells. **a** Immunofluorescence of Ki67 and Caspase7 in spheroids from oral mucosa- and skin-derived cells. A larger number of Ki67-positive cells were observed in the spheroids with additives than those in the spheroids without additives regardless of the origins. The results from immunofluorescence for Caspase 7 showed positive cells in oral mucosal spheroids without additives but only a smaller number of faintly positive cells were noted in the spheroids with additives. The spheroids from the skin showed faint staining only in the spheroids without additives but no positive cells in the spheroids with additives. **b** The expression of Ki67 and Caspase7 in spheroids from oral mucosa- and skin-derived cells was analyzed using qRT-PCR, and the levels in spheroids with or without additives were compared. Higher expression of Ki67 was observed in both oral mucosal spheroids and skin-derived spheroids with additives than that without additives. On the other hand, the expression of Caspase 7 was significantly higher in the spheroids without additives than that with additives. **c** The expression levels of Caspase7 in spheroids from oral mucosa- and skin-derived cells were compared. The expression of Caspase7 was significantly higher in the spheroids from oral mucosa-derived cells than in the spheroids from skin-derived cells regardless of the additives. Scale bars = 50 μm. DAPI, 4′,6-diamidino-2-phenylindole. The data are presented as the mean ± SEM; *n* = 3. Scale bars = 50 μm *p < 0.05; **p < 0.01; ***p < 0.001

A larger number of Ki67-positive cells were observed in the spheroids with additives than in the spheroids without additives either in the spheroids from oral mucosa- and skin-derived cells (Fig. 3a). The Caspase 7-positive cells were observed in the oral mucosal spheroids without additives but only a smaller number of faintly positive cells with additives. The spheroids from the skin showed faint Caspase 7-positive cells in the spheroids without additives, but no positive cell was observed in the spheroids with additives (Fig. 3a).

The expressions of proliferation and apoptosis genes were analyzed using qRT-PCR. The expression of Ki67 was higher in the spheroids cultured with the additives than in the spheroids without the additives (Fig. 3b). On the other hand, the expression of Caspase7 was significantly higher in the spheroids cultured without the additives than in the spheroids with the additives (Fig. 3b). When the expression of Caspase7 was compared between the spheroids from oral mucosa-derived cells and the spheroids from skin-derived cells, the expression was significantly higher in the spheroids from oral mucosa-derived cells regardless of the presence of additives (Fig. 3c).

### Comparison of stem cell marker expression in spheroids from oral mucosa- and skin-derived cells

**Fig. 4:**
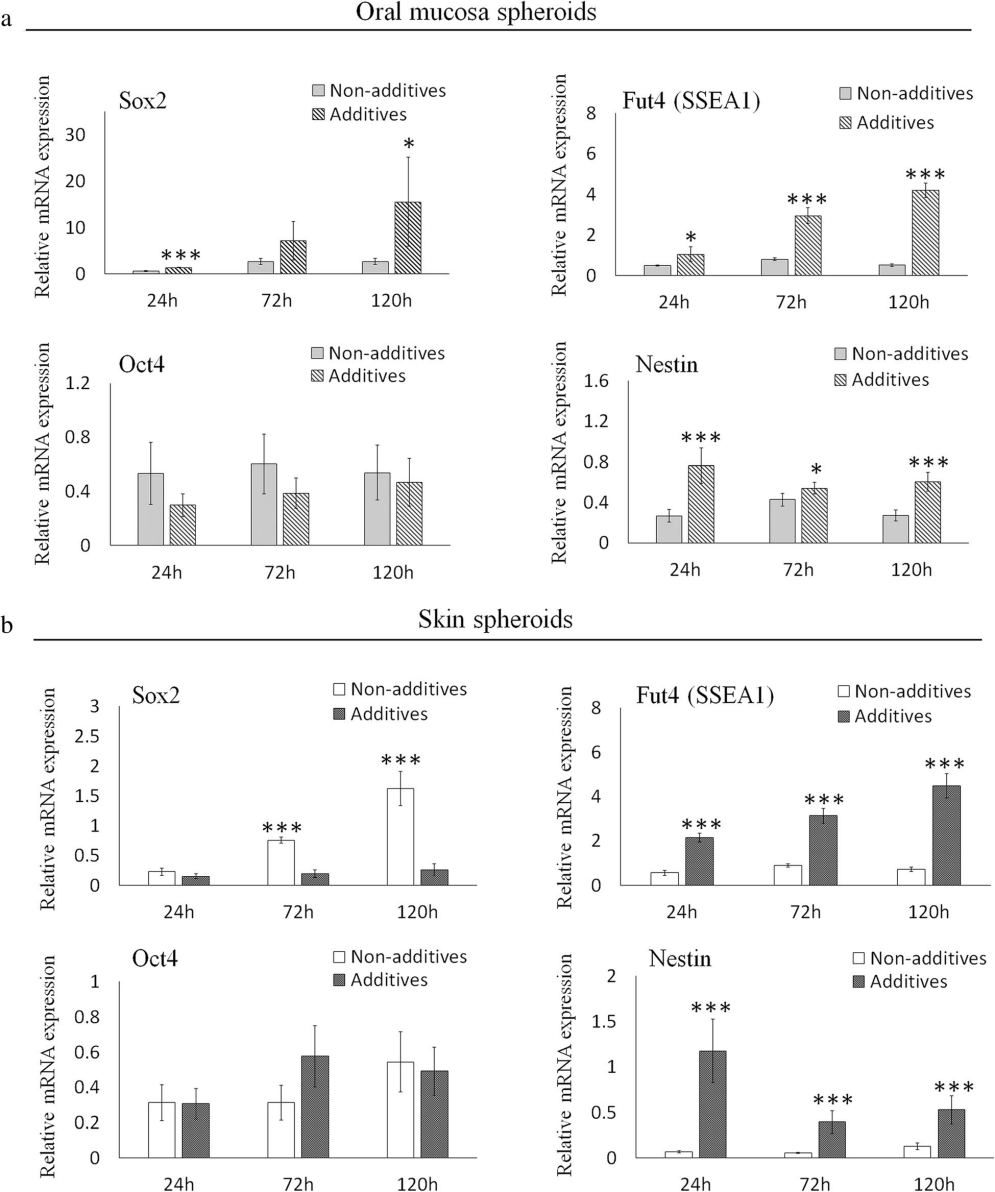
Effect of bFGF, EGF, and B27 supplement on stem cell marker expression in spheroids from oral mucosa- and skin-derived cells. **a** The expression of Sox2, Fut4 (SSEA1), Oct4, and Nestin in spheroids from oral mucosa-derived cells was analyzed using qRT-PCR, and the levels in spheroids cultured with or without the additives were compared. **b** The expression of Sox2, Fut4 (SSEA1), Oct4, and Nestin in the spheroids from skin-derived cells was analyzed using qRT-PCR, and the levels in spheroids cultured with or without the additives were compared. The data are presented as the mean ± SEM; *n* = 3. *p < 0.05; **p < 0.01; ***p < 0.001

The expression of pluripotency-associated genes was analyzed using qRT-PCR. In spheroids from oral mucosa-derived cells, the expression of Sox2, Fut4 (SSEA1), and Nestin were higher in spheroids cultured with the additives than in those without additives, while the expression of Oct4 was not affected by the presence of the additives (Fig. 4a). In spheroids from skin-derived cells, the expression of Fut4 (SSEA1) and Nestin was significantly higher when the spheroids were cultured with the additives (Fig. 4b). On the other hand, the expression of Sox2 dropped in the presence of the additives. Similar to the oral mucosa spheroids, the expression of Oct4 showed no significant difference with or without the additives.

**Fig. 5:**
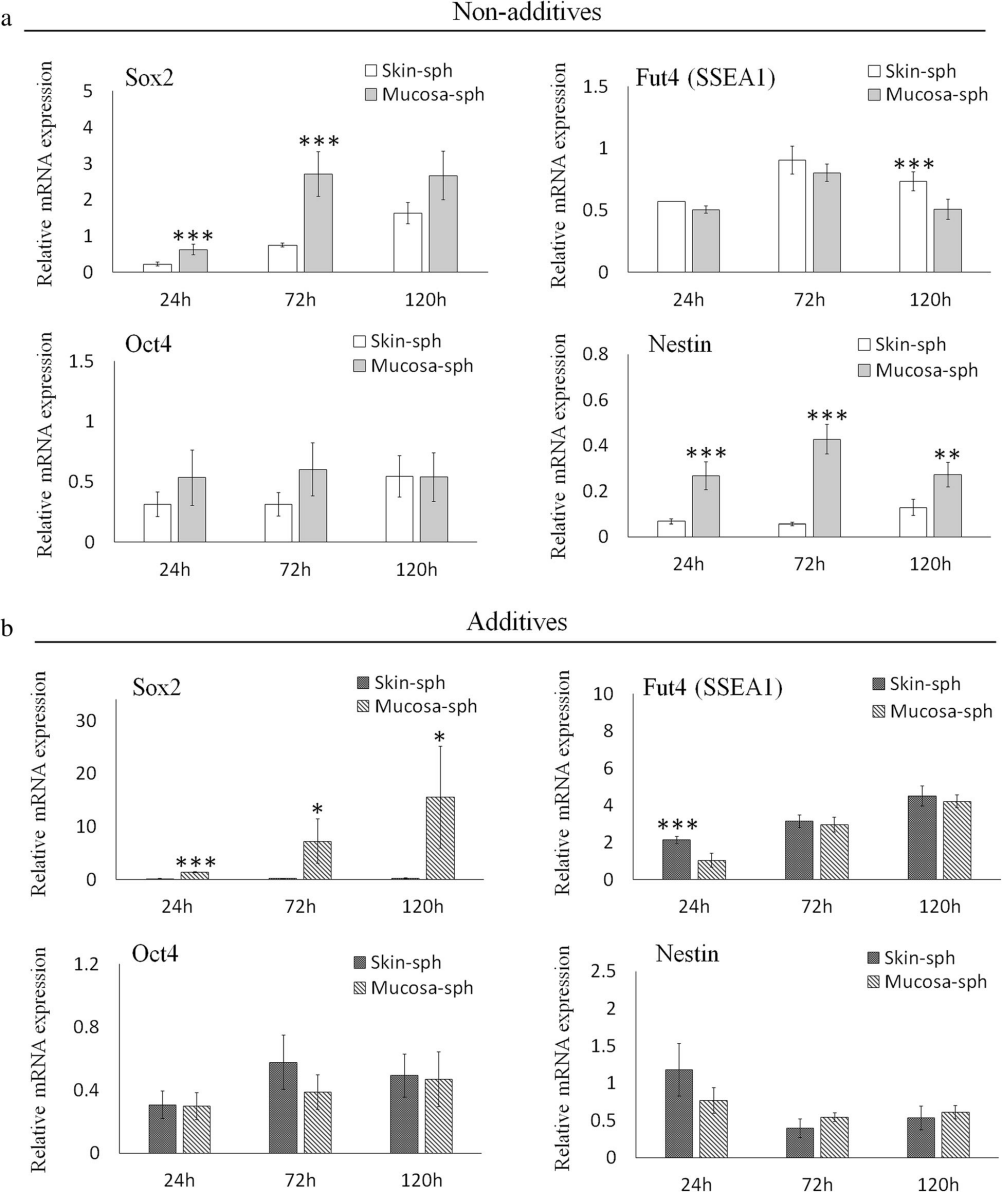
Direct comparison of stem cell marker expression between spheroids from oral mucosa- and skin-derived cells. **a** The expression levels of Sox2, Fut4 (SSEA1), Oct4, and Nestin in spheroids from oral mucosa- and skin-derived cells without bFGF, EGF, and B27 supplement were analyzed using qRT-PCR and compared. **b** The expression levels of Sox2, Fut4 (SSEA1), Oct4, and Nestin in spheroids from oral mucosa- and skin-derived cells cultured with bFGF, EGF, and B27 supplement were analyzed using qRT-PCR and compared. The data are presented as the mean ± SEM; *n* = 3. *p < 0.05; **p < 0.01; ***p < 0.001

Next, stem cell marker expression was compared between oral mucosa- and skin-derived cell spheroids. The expression of Sox2 was higher in spheroids from oral mucosa-derived cells than in those from skin-derived cells, and the tendency was not affected by the presence of additives (Fig. 5a, b). The expression of Nestin was higher in spheroids from oral mucosa-derived cells than in those from skin-derived cells without the additives (Fig. 5a). However, this difference was not observed when the spheroids were cultured with the additives (Fig. 5b). The expression of Fut4 (SSEA1) in spheroids from oral mucosa-derived cells was lower than that in skin-derived cell spheroids at 120 h without additives and at 24 h with additives, but the difference was not observed under other conditions. The expression of Oct4 showed no significant difference between the spheroids from oral mucosa- and skin-derived cells, and this tendency was not affected by the presence of additives.

### Comparison of neurogenic differentiation capability between spheroid-derived cells from the oral mucosa and skin

**Fig. 6:**
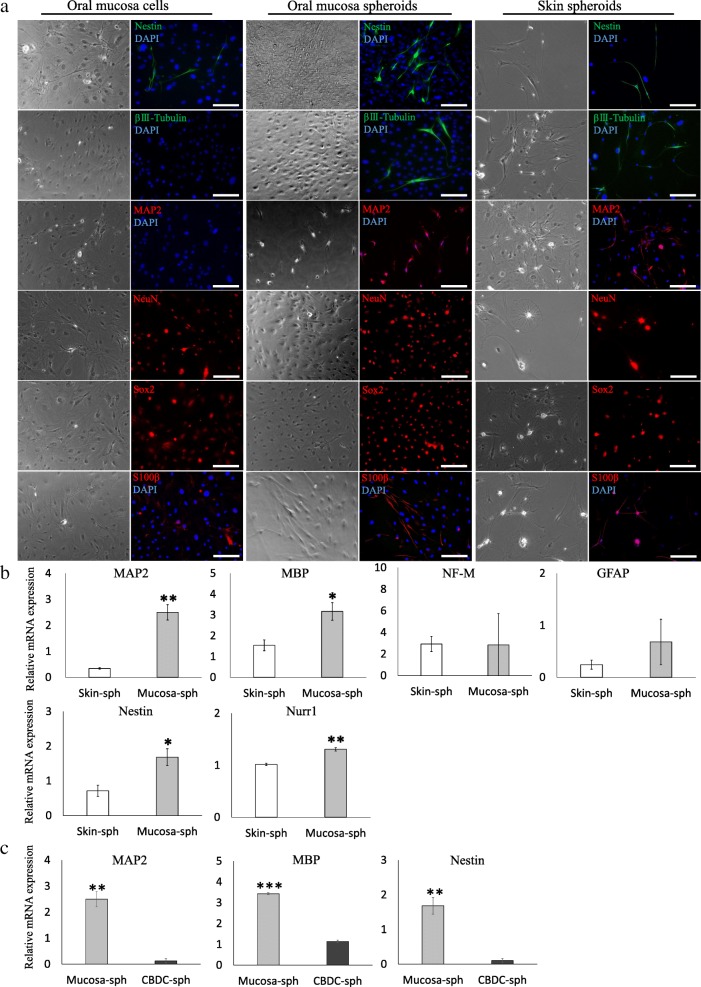
Neurogenic induction of spheroid-derived cells from oral mucosa and skin. **a** Immunofluorescence images. βIII-tubulin- and MAP 2-positive cells were not observed, while Nestin-, NeuN-, Sox2-, and S100β-positive cells were found in oral mucosa cells (two-dimensional culture). Nestin-, βIII-tubulin-, MAP 2-, NeuN-, Sox2-, and S100β-positive cells were observed in the spheroid-derived cells from both oral mucosa and skin. **b** The expression levels of neural cell markers were analyzed using qRT-PCR. The expression levels of MAP 2, MBP, Nestin, and Nurr1 in spheroid-forming cells from the oral mucosa were significantly higher than those in spheroid-forming cells from the skin. **c** Expression of MAP 2, MBP, and Nestin in the spheroid-forming cells from the oral mucosa and CBDCs. The expression levels of MAP 2, MBP, and Nestin in the spheroid-forming cells from oral mucosa were significantly higher than those in spheroid-forming cells from CBDCs. The data are presented as the mean ± SEM; *n* = 3. *p < 0.05; **p < 0.01; ***p < 0.001. Scale bars = 50 μm. DAPI, 4′,6-diamidino-2-phenylindole

Two-dimensionally cultured oral mucosal cells contained some cells positive for Nestin, NeuN, Sox2, and S100β, but they were negative for βIII-tubulin and MAP 2 (Fig. 6a). The spheroid-derived cells from both the oral mucosa and skin were positive for Nestin, βIII-tubulin, MAP 2, NeuN, Sox2, and S100β (Fig. 6a). To investigate the level of differentiation, the mRNA expression levels of MAP 2, MBP, NF-M, GFAP, and Nestin were analyzed. Furthermore, the expression of a specific dopaminergic neuron marker Nurr1 was also assessed by qRT-PCR (Fig. 6b). The expression of MAP 2, MBP, Nestin, and Nurr1 mRNA in oral mucosal spheroid-derived cells was significantly higher than that in skin spheroid-derived cells. The expression level of NF-M was not different between spheroid-derived cells of both origins. The expression of GFAP in the oral mucosal spheroid-derived cells was higher than that in the skin spheroid-derived cells, but the difference was not significant due to large variations.

Although the relatively higher expression of several neuronal differentiation markers was observed in the spheroids from oral mucosa-derived cells, it was not clear if the level is comparable to those in other somatic stem cells such as mesenchymal stem cells. Accordingly, we next compared the expression levels of MAP 2, MBP, and Nestin between the spheroids from oral mucosa-derived cells and the spheroids from CBDCs which is known as a superior source of mesenchymal stem cells in mouse and can differentiate into neurogenic cell lineages [22–24]. After neurogenic induction, the expression of MAP 2, MBP, and Nestin in the oral mucosal spheroid-derived cells were exceedingly higher than those in the spheroid-derived cells from CBDCs (20.08-, 3.02-, and 16.18-fold, respectively) (Fig. 6c).

## Discussion

### Characteristics of spontaneous spheroids from oral mucosa-derived cells

During mechanical spheroid formation, ideally, almost all cells in the culture participate in spheroid formation. Thus, the process is not selective [25]. On the other hand, spontaneous spheroid formation occurs under static conditions. It starts only from the cells that can survive without attachment, and the cells should have an ability to proliferate to form a spheroid. Accordingly, spontaneous spheroid formation is selective but not as efficient as mechanical spheroid formation. In fact, skin-derived precursors (SKPs), which are well-known to spontaneously form spheroids, can be obtained from embryonic tissue and are difficult to obtain from adult tissue [26]. Our method for spontaneous spheroid formation can overcome this limitation, because spheroid formation is feasible even with cells cultured through 4–5 passages (Additional file 1: Figure S1). It is also important to ask if the spheroids formed using our method are identical to spontaneously formed spheroids, such as SKP spheroids. The spontaneously formed spheroids from oral mucosa-derived cells were positive for Sox2, SSEA1, Oct4, Nanog, and Nestin and capable of neurogenic differentiation, which indicates that they exhibited stemness characteristics identical to those of previously reported spontaneous spheroids [2, 12].

One of the fundamental differences between the current and previously reported spontaneous spheroid formation procedures is the culture medium contents. It has been reported that spontaneous spheroid formation requires a serum-free environment and the addition of growth factors, such as bFGF, EGF, and B27 supplement [27, 28]. On the other hand, spontaneous spheroid formation induced by our method can be achieved in a serum-containing environment without those additives. Rather, serum was essential for the cell-to-cell attachment in spontaneous spheroid formation. The reason why SKPs do not require serum is not clear. The SKPs were obtained from dissociated cells without culture. Because noncultured cells were used, the cells may still retain some essential factors for the initial step in spheroid formation. Alternatively, the mechanism of spheroid formation in our approach is completely different from the method applied for SKP culture. This point should be clarified in future experiments.

### Role of additives for spontaneous spheroid formation and maintenance

With the additives, the number of spheroids at 24 h was approximately two times larger than that without the additives, which indicates some roles of the additives in spheroid formation. Since spontaneous spheroid formation was feasible without the additives, they facilitate spheroid formation but are not essential. On the other hand, without the additives, spheroid size and number decreased over time. This tendency was more evident in the spheroids derived from oral mucosa-derived cells than in those from skin-derived cells. This indicates that those factors are indispensable for the maintenance of spontaneously formed spheroids, and their effect was larger on spheroids from oral mucosa-derived cells.

To confirm this, we focused on a cell-proliferating marker Ki67 and Caspase7 as an apoptosis marker, and the expression pattern and the level were compared in the spheroids with or without the additives. The spheroids with additives showed a larger number of Ki67-positive cells than the spheroids without additives regardless of the origins, which was further confirmed by the higher expression of Ki67. This indicates the proliferative effect of those additives for spheroid-forming cells. Furthermore, the expression levels of Caspase 7 were significantly higher in the spheroids without additives, which support our hypothesis that the additives may be essential to maintain the stem cells in spheroids and the lack of additives results in apoptosis of the spheroid-forming cells. Finally, we compared the expression of Caspase 7 between spheroids from skin- and oral mucosa-derived cells. The higher expression of Caspase7 in the spheroids from oral mucosa-derived cells may partly explain the significantly smaller size and number of spheroids in oral mucosa-derived cells at 120 h.

Though the detailed mechanism of spheroid maintenance is beyond the scope of this study, one possible explanation is the function of bFGF, which contributes to the pluripotency maintenance and reduces stress-induced apoptosis in human embryonic stem cells [29]. It is possible that similar mechanisms are applied in the maintenance of stemness in spheroid-forming cells, increasing their lifespan and maintaining them in a relatively undifferentiated state [16]. The results of this study showed that the factors required for spheroid formation are not identical to those necessary for spheroid maintenance, and this should be kept in mind when discussing the requirement of these additives. Because the period previously reported for spontaneous spheroid formation is longer than ours (2–3 weeks vs. 24 h), the roles of the additives in spheroid formation and maintenance could not be well distinguished [26].

### Similarities and differences in stem cell marker expression between spontaneously formed spheroids from oral mucosa- and skin-derived cells

In the spheroids from oral mucosa-derived cells, the expression of Sox2, Fut4 (SSEA1), and Nestin was higher with the additives, while the level of Oct4 expression was not affected. The lower expression levels of Sox2, Fut4 (SSEA1), and Nestin in the absence of the additives may correspond to an unhealthy status of the cells because those factors play important roles in the maintenance of spheroids. Oct4, a transcription factor encoded by the Pou5fl gene, is critical for the self-renewal and maintenance of stemness properties of embryonic stem cells [30]. Although the expression level of Oct4 should have decreased in a manner corresponding with the unhealthy status of the cells, as was observed with the other markers, it is known that Oct4, in association with HIF-1α, has other roles in survival, proliferation, and differentiation of damaged cells [31]. It is conceivable that dying or unhealthy cells express Oct4 via this distinct pathway, which may compensate for the reduction in basal Oct4 activity. Further study will be needed to clarify this mechanism.

In the spheroids from skin-derived cells, the effect of the additives on the expression of stem cell marker genes was almost identical to that in the spheroids from oral mucosa-derived cells, except for Sox2, which showed relatively higher expression without the additives. The expression of Nanog, Oct4, and Sox2 is regulated by LIF. However, a previous study showed an alternative regulatory pathway for Sox2, which is also a critical downstream target of fibroblast growth factor (FGF) signaling [32]. There might be a difference in the regulatory mechanisms of those two or other pathways in mucosa- and skin-derived stem cells that contributed to the differential gene expression profile.

In the current study, a direct comparison of spheroids from oral mucosa- and skin-derived cells was performed. Despite our expectation, the expression levels of most of the tested stem cell markers were identical except for Sox2. The expression of Nestin was significantly higher in spheroids from oral mucosa-derived cells than in those from skin-derived cells. However, the difference was not observed when the spheroids were cultured with additives, which indicates that the difference is not fundamental. The reason for the difference in Sox2 expression between the oral mucosa- and skin-derived cell spheroids is not clear from this study. Sox2 is a self-renewal maker shared by both embryonic stem cells and neural stem cells [33]. It has been shown that the lamina propria of the adult oral mucosa harbors a primitive stem cell population with a distinct neural crest-like phenotype [16]. Sox2 is localized in these neural crest-like cells, and the cells are a possible source of spheroid-forming cells. Although Sox2-positive neural crest precursor cells also exist in mammalian skin, the higher expression of Sox2 in spheroids from oral mucosa-derived cells might be a reflection of their abundance in oral mucosa [34].

### Neural differentiation of spontaneous spheroid-forming cells from oral mucosa

After neurogenic induction, the monolayer culture of oral mucosal cells failed to express neural markers, such as MAP 2 and βIII-tubulin. The presence of these differentiated neural cell markers in spheroid-derived cells suggests improved neurogenic differentiation capability of spheroid-forming cells. Since spheroids from both oral mucosa- and skin-derived cells contained cells positive for Nestin, βIII-tubulin, MAP 2, NeuN, Sox2, and S100β, the relative expression of neurogenic markers was compared using qRT-PCR. Among the tested markers, the expression levels of MAP 2, MBP, and Nestin were significantly higher in spheroids from oral mucosa-derived cells than in spheroids from skin-derived cells. In addition, higher expression of a dopaminergic neuron marker Nurr1 in spheroids from oral mucosa-derived cells was observed. Taken together, these findings suggest an advantage of using these oral mucosa cells for the treatment of neurodegenerative disorders such as Parkinson disease. More importantly, the expression of MAP 2, MBP, and Nestin were exceedingly higher in spheroids from oral mucosa-derived cells than in spheroids from mesenchymal stem cells from CBDCs. This might be due to the presence of neural crest-derived stem/progenitor cells in oral mucosa-derived spheroids and further strengthen the advantage of this unique cell source for the application to neurodegenerative disorders.

However, one of the limitations of this study was the lack of in vivo experiments. The results from in vitro study may not always match to that of in vivo experiments and further study using an appropriate animal model should be performed in the future.

## Conclusions

The results from this study showed that the spontaneous spheroid formation was feasible with oral mucosa-derived cells using our method. Although the spontaneous spheroid formation was feasible without bFGF, EGF, and B27 supplement, they improved the efficiency of spheroid formation and more importantly, were essential for the spheroid maintenance. Spontaneous spheroids from oral mucosa-derived cells contain highly potent stem cells, which were as good as skin-derived stem cells. The high expression of certain neuronal marker genes suggests an advantage of these cells for regeneration therapy for neuronal disorders.

## Additional file

Additional file 1: **Figure S1.** Phase-contrast images of spontaneous spheroids from skin-derived cells. Note the presence of spontaneously formed spheroids even from passaged cells. (A) Spontaneous spheroid formation from skin-derived cells as passage 2. (B) Spontaneous spheroid formation from skin-derived cells at passage 3. (C) Spontaneous spheroid formation from skin-derived cells at passage 4. (D) Spontaneous spheroid formation from skin-derived cells at passage 5. (PPTX 564 kb)

## Data Availability

Contact the corresponding author for availability.

## Abbreviations

bFGF: Basic fibroblast growth factor
CBDCs: Compact bone-derived cells
EGF: Epidermal growth factor
FBS: Fetal bovine serum
qRT-PCR: Quantitative reverse transcription polymerase chain reaction
