# Supplementary material for: Characterization of spontaneous spheroids from oral mucosa-derived cells and their direct comparison with spheroids from skin-derived cells
Source: Stem Cell Res Ther. 2019 Jun 24;10:184. doi: 10.1186/s13287-019-1283-0 (PMC6591807; doi:10.1186/s13287-019-1283-0)
Supplement: Supplementary file 1 — Figure S1. Phase-contrast images of spontaneous spheroids from skin-derived cells. Note the presence of spontaneously formed spheroids even from passaged cells. (A) Spontaneous spheroid formation from skin-derived cells as passage 2. (B) Spontaneous spheroid formation from skin-derived cells at passage 3. (C) Spontaneous spheroid formation from skin-derived cells at passage 4. (D) Spontaneous spheroid formation from skin-derived cells at passage 5. (PPTX 564 kb) [file 13287_2019_1283_MOESM1_ESM.pptx]

## Slide 1
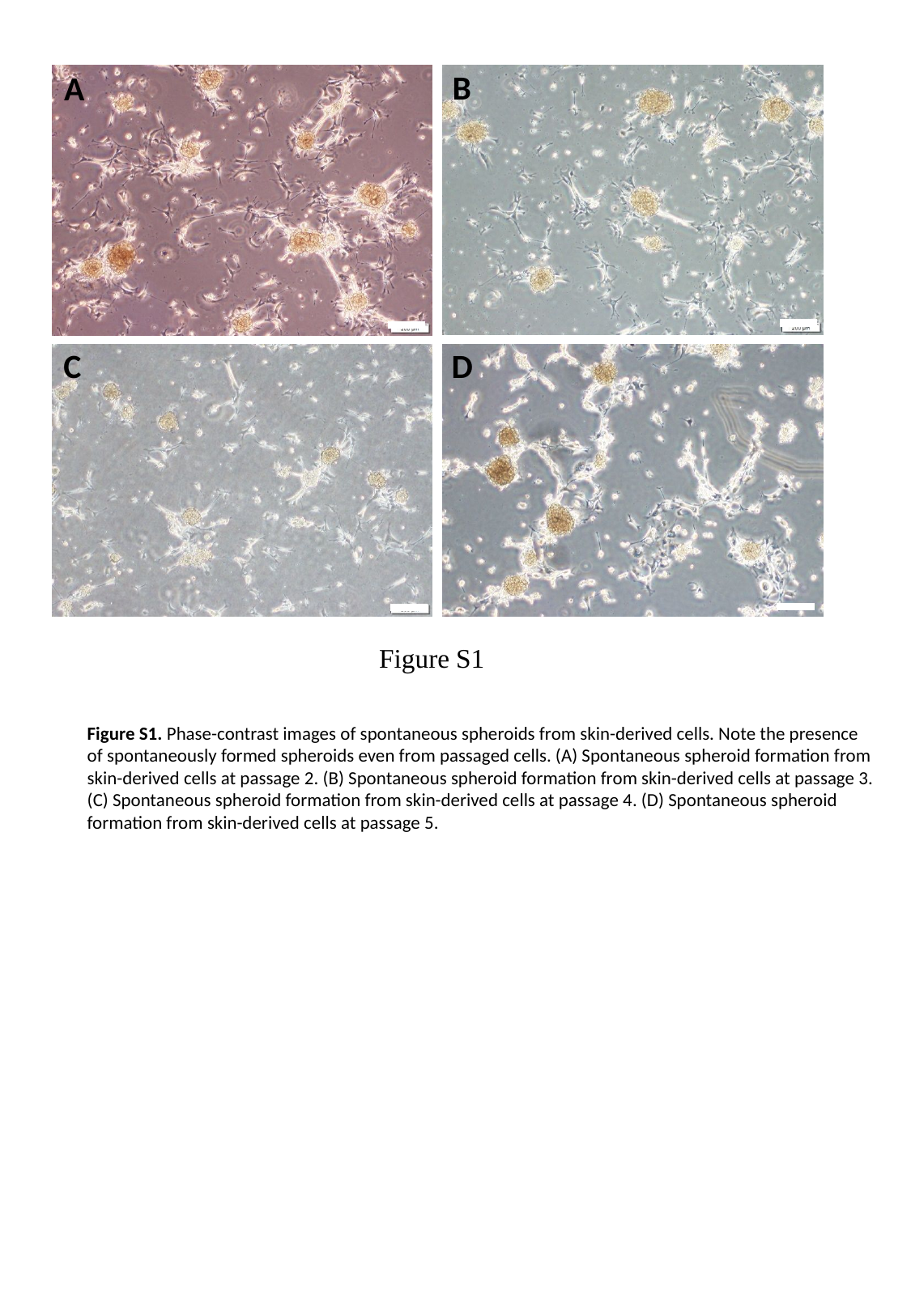

B
A
D
C
Figure S1
Figure S1. Phase-contrast images of spontaneous spheroids from skin-derived cells. Note the presence of spontaneously formed spheroids even from passaged cells. (A) Spontaneous spheroid formation from skin-derived cells at passage 2. (B) Spontaneous spheroid formation from skin-derived cells at passage 3. (C) Spontaneous spheroid formation from skin-derived cells at passage 4. (D) Spontaneous spheroid formation from skin-derived cells at passage 5.
